# Matrix Metalloproteinase Inhibition in Melanoma

**DOI:** 10.1111/exd.70203

**Published:** 2026-01-16

**Authors:** Ellie Zhang, Varsha Thakur, Barbara Bedogni

**Affiliations:** ^1^ Dr. Phillip Frost Department of Dermatology and Cutaneous Surgery, Miller School of Medicine University of Miami Miami Florida USA

**Keywords:** extracellular matrix, matrix metalloproteinase inhibitors, matrix metalloproteinases, melanoma

## Abstract

Matrix metalloproteinases (MMPs) are involved in the degradation of the extracellular matrix (ECM) and are found to participate in all stages of tumour progression including modifying signalling pathways, regulating cytokines and promoting tumour growth, particularly by inducing angiogenesis and facilitating cancer spread. Extensive research has been concentrated on identifying and developing MMP inhibitors for cancer treatment, including melanoma, with particular focus on MMP‐2, MMP‐9 and MMP‐14. MMP‐2 and MMP‐9 are gelatinases involved in collagen degradation, tumour invasion and angiogenesis, while MMP‐14 activates other MMPs and promotes tumour cell migration. Early broad‐spectrum MMP inhibitors showed limited success and significant side effects. However, selective MMP inhibitors offer a more targeted approach that may address these problems. By focusing on specific MMPs essential for melanoma invasion, metastasis and angiogenesis, these inhibitors have the potential to improve treatment efficacy and reduce the off‐target effects seen with earlier broad‐spectrum therapies. Recent years have seen a marked increase in studies on natural MMP inhibitors for melanoma, driven by their biocompatibility and reduced side effects. In addition to inhibiting MMPs, many of these inhibitors also provide antioxidant, anti‐inflammatory and immune‐modulatory benefits, thus enhancing their therapeutic potential and overall effectiveness in cancer treatment. These findings highlight the promising role of MMP inhibitors in melanoma therapy, suggesting a shift towards more targeted and combinatory treatment strategies. This review aims to provide an up‐to‐date overview of the advancements and therapeutic prospects of both synthetic and natural MMP inhibitors in melanoma treatment.

## 1 Introduction

The extracellular matrix (ECM) is a complex network of proteins and carbohydrates that surrounds cells and supports and provides structure to cells and tissues in the body [1]. ECM helps cells attach to and communicate with nearby cells. It plays a vital role in the regulation of cell–cell interactions and in the maintenance of tissue homeostasis. The homeostasis of ECM is maintained through the tightly regulated and precisely coordinated process involving the production, deposition and turnover of its components [2]. Abnormal structural changes in ECM may lead to the development of certain diseases, such as cancer [3, 4]. In cancers, the synthesis, modification and degradation dynamics of ECM components are deregulated, resulting in changes to composition, density and mechanical properties [2, 5, 6]. As a dynamic and critical component of the tumour microenvironment (TME), ECM has gained increasing recognition as a pivotal feature in tumour biology through promoting various hallmarks of cancer via biochemical and biomechanical signalling pathways [7, 8].

Melanoma is a type of skin cancer that arises from melanocytes, the pigment‐producing cells in the skin. Melanoma is the most severe form of skin cancer characterised by a poor prognosis due to its aggressive nature, high potential for spreading and multiple gene mutations with high intra‐tumour and inter‐tumour molecular heterogeneity [9]. Despite recent advances in therapy, the survival rate for metastatic melanoma is < 10% [10, 11]. Among the mutations in melanoma, the most frequently observed are the ones with the presence of the BRAF proto‐oncogene serine/threonine‐protein kinase B‐raf (BRAF) or the NRAS proto‐oncogene GTPase (NRAS) activating mutations—accounting for about 50% and 20%–25% of melanomas, respectively [12, 13, 14]. Approximately 80% of BRAF mutations are accounted for by the BRAFV600E mutation where the valine 600 is substituted with the glutamic acid (p.V600E) [13]. This mutation leads to the over‐expression and hyperactivation of BRAF [15]. The molecular composition of the tumour and the tumour microenvironment significantly influences the progression of melanoma. Facilitated by the expression of proteases that degrade the ECM, melanoma cells are able to invade the surrounding microenvironment both locally and at a distance from the primary tumour [7, 16].

Matrix metalloproteinases (MMPs) are a family of 24 endopeptidases containing a catalytic zinc ion site that plays an important role in the degradative and reconstructive processes of the ECM [17, 18, 19, 20, 21]. Based on substrate specificity or proteolytic function, they are classified as several groups: collagenases (MMP‐1, MMP‐8, MMP‐13), gelatinases (MMP‐2, MMP‐9), stromelysins (MMP‐3, MMP‐10, MMP‐11), matrilysins (MMP‐7, MMP‐26), metalloelastase (MMP‐12), membrane‐type MMPs (MMP‐14, MMP‐15, MMP‐16, MMP‐17, MMP‐24 and MMP‐25) and other MMPs (MMP‐19, MMP‐20, MMP‐21, MMP‐23, MMP‐27 and MMP‐28) [18, 22].

Primarily associated with ECM degradation, they also participate in different biological and physiological processes and play essential roles in cell behaviours, including proliferation, migration, differentiation, angiogenesis, apoptosis and host defence [23]. Under normal physiological conditions, the activities of MMPs are precisely regulated at multiple levels, including transcription, activation of precursor zymogens, interaction with specific ECM components and inhibition by endogenous inhibitors [20, 24, 25]. A key feature of MMPs is the presence of a methionine residue in the active centre and the use of zinc in the enzymatic reaction [26].

MMPs are regulated by the secretion of pro‐MMPs in an inactive form and their inhibition by endogenous protein inhibitors [27]. They are secreted as zymogens, which are inactive due to the interaction between the zinc ion and a cysteine residue. The removal of this interaction, known as the ‘cysteine switch’, that can occur either after secretion or intracellularly by pro‐hormone convertases such as furin, results in partial activation of MMPs [17]. Complete activation is achieved when the proteinase cleaves its pro‐domain, a process known as autocatalysis [17].

Additionally, the balance between the degradative and reconstructive processes of MMPs is regulated by tissue inhibitors of metalloproteinases (TIMPs). TIMPs are a class of endogenous protein inhibitors that inhibit MMPs by reversibly binding to the MMP catalytic site in a stoichiometric manner [17, 19]. This delicate balance between MMPs and TIMPs maintains ECM homeostasis [28].

Overexpression of MMPs or insufficient regulation by TIMPs results in various diseases including cancers such as melanoma [22, 27]. MMPs are found to participate in all stages of tumour progression, impacting various biological functions. They modify signalling pathways, regulate cytokines and promote tumour growth, particularly by inducing angiogenesis and facilitating cancer spread [19, 29]. High levels of MMPs are strongly correlated with metastatic spread and poor prognosis of melanoma, as they perform several functions that favour tumour progression, including the degradation of surrounding tissues, and the modulation of growth factors, membrane receptors, inflammatory proteins, adhesion molecules and chemo‐attractive proteins [7, 17, 18, 30].

MMPs have long been regarded as potential therapeutic targets for cancer treatment due to their distinct involvement in various stages of tumour progression, such as tumour invasion, neoangiogenesis and metastasis. Extensive research efforts have concentrated on identifying and developing MMP inhibitors for cancer therapy. These inhibitors hold promise as both biomarkers and therapeutic targets for managing melanoma patients. This review aims to provide an up‐to‐date summary of MMP inhibitors and their potential therapeutic implication for melanoma patients.

## 2 Development of MMP Inhibitors

MMP inhibitors (MMPIs) are the molecules that interact with MMPs and modulate their functions. They can be classified into synthetic inhibitors, endogenous inhibitors and natural inhibitors [22, 31].

### 2.1 Synthetic Inhibitors

In 1988, researchers developed the first synthetic MMPI (SC‐44463) using a hydroxamic acid compound to block collagenase, thereby preventing metastasis in a mouse model. This breakthrough marked the beginning of the era of MMP inhibition therapeutics [32]. Four generations of synthetic MMPIs have been developed so far, with each generation having an affinity for different binding sites on MMPs [22]. Clinical trials of the synthetic MMPI were performed on various types of cancer treatment [33, 34, 35] during the late 1990s and early 2000s. Nevertheless, all trials encountered failure primarily due to the low specificity and significant musculoskeletal side effects of MMPIs.

The first generation of MMPIs are hydroxamate based inhibitors which have the capability to inhibit a broad spectrum of MMPs [36]. They are primarily small molecules or peptides that contain the hydroxamate zinc‐binding group (‐CONHOH), which exhibits strong affinity with the Zn^2+^ ion on the catalytic domain of MMPs [37]. Clinical trials of this type of MMPIs on cancer treatment include Batimastat (BB‐94) [38], Marimastat (BB‐2516) [39, 40], MMI270 (CGS27023A) [41, 42] and Prinomastat (AG3340) [43, 44]. Batimastat was the first MMPI to enter clinical trial for malignant tumours by inhibiting MMP‐1, MMP‐2, MMP‐7 and MMP‐9 [38]. However, due to its poor oral bioavailability, it was later replaced by Marimastat [39]. Prinomastat was tested for treating advanced cancer and oesophagus cancer due to its high affinity for MMP‐2, MMP‐3, MMP‐9, MMP‐13 and MMP‐14 [43, 44]. Unfortunately, all of these studies failed due to lack of specificity, efficacy and severe side effects [45].

The second generation of MMPIs were developed with the intention of addressing the issues associated with the first generation MMPI. They are non‐hydroxamate based inhibitors and the zinc‐binding groups in the complex of Zn^2+^ are thiolate, carboxylate and phosphinate [22]. The non‐hydroxamate group has a relatively weak chelating affinity to the Zn^2+^, allowing the molecule greater freedom of orientation compared to hydroxamate‐based MMPIs. Consequently, this reduced side effects to some extent [46]. Clinical trials of this type of MMPIs on tumour therapy include Tanomastat (Bay 12‐9566) [33, 47, 48], Metastat (COL‐3) [49, 50], Rebimastat (BMS‐275291) [51, 52, 53] and S‐3304 [54]. All these trials exhibited good tolerability; however, efficacy was poor.

The third generation MMPIs were developed to address the limitations observed in both the first and second generations. It specifically targets the catalytic domain of MMPs which do not rely on zinc‐binding groups. The catalytic domain contains many recognition pockets (named S_n_ and S_n_′) that the inhibitors can fit into. Due to variations in the depth of these pockets, they have different selectivity towards MMPIs [22, 55, 56]. This distinction enables the development of more selective MMPIs with high inhibition capacity as summarised by Li et al. [22, 57, 58, 59, 60].

We previously reported that the thiirane derivative ND‐322 effectively inhibited melanoma tumour growth and metastasis in a mouse model [61]. ND‐322 exhibited high selectivity for gelatinases (MMP2 and MMP9) and MT1‐MMP through a mechanism‐based inhibition. In this process, the glutamate in the active site of these proteinases facilitates a reaction that opens the thiirane ring to the corresponding thiolate and results in picomolar tight‐binding inhibition [62] and long residence times of the inhibitor on the target proteinases; for instance, ND‐322 has a residence time of 23.4 min with MMP2, significantly longer than the residence times of MMP2 with TIMP1 (6.9 min) or TIMP2 (6.7 min) [63]. This specific inhibition mechanism underpins the selectivity of ND‐322 for gelatinases and MT1‐MMP, with minimal effect on other MMPs [64].

As the full‐length structures of MMPs are revealed over time, the roles of other domains within distal structures, such as the hemopexin domain, the collagen binding domain and the pro‐peptide domain, in signal transmission, protein–protein interactions and substrate recognition become increasingly understood. The fourth generation MMPIs were designed to target these non‐catalytic domains, aiming to disrupt protein–protein interactions and impacting MMP function beyond catalysis for highly selective and potent inhibitors [65, 66]. For example, a small molecule NSC405020 was reported to selectively bind to the hemopexin domain of MMP‐14, inhibiting MMP‐14 homodimerization and the interaction between the hemopexin domain and the catalytic domain. As a result, it prevents the degradation of type I collagen and effectively halts tumour growth [67]. Scannevin et al. [68] identified a highly selective compound named JNJ0966, which targets the pro‐peptide domain of MMP‐9. This compound effectively inhibits the activation of MMP‐9 zymogen while sparing other MMPs from its effects [68].

### 2.2 Endogenous Inhibitors

The primary endogenous inhibitors that regulate the activities of MMPs are α‐2 macroglobulin and TIMPs [18, 28]. In the fluid phase, MMP activities are primarily regulated by α‐2 macroglobulin, a plasma glycoprotein that binds to MMPs, forming an inactive complex that is rapidly cleared by receptor‐mediated endocytosis [28]. It was found that the levels of the α‐2 macroglobulin receptor (LRP/α2‐MR) were less expressed in the invasive sub‐clones of the A2058 melanoma cell line compared to the non‐invasive ones, supporting the hypothesis that the down‐regulation of the LRP/α2‐MR complex can increase tumour cell invasiveness [69].

TIMPs are a class of endogenous protein inhibitors consisting of a family of four genes: TIMP1‐4 [70]. They form a 1:1 complex with MMPs in a non‐selective manner by reversibly binding to the MMP catalytic site [17, 18]. Although TIMPs can interact with different MMPs, MMP‐1 is not targetable by TIMP‐1 [18, 28]. Additionally, TIMPs have inhibitory preferences, such as TIMP‐1 favouring the inhibition of MMP‐9 [18]. The delicate balance between MMPs and TIMPs maintains ECM homeostasis, and disruptions in this balance can lead to various diseases [22, 27]. Studies have shown that dysregulation of TIMPs can influence melanoma progression. For instance, TIMP‐1 overexpressing cells exhibited a significant reduction in the metastatic potential of B16‐F10 cells in mice [71, 72]. Similarly, B16‐F10 cells in mice transfected with TIMP‐2 showed reduced angiogenesis and decreased cell migration and invasion [73]. However, TIMP‐2 overexpressing clones were found to be more resistant to apoptosis than parental and control melanoma cells, indicating a potential role for TIMP‐2 in cell survival [73]. A study by Das et al. [74] examining TIMP‐3 expression in patients with stage I‐IV melanoma found that TIMP‐3 expression decreased with melanoma progression. This suggests that TIMP‐3 functions as a tumour suppressor in melanoma and negatively regulates aspects of the metastatic cascade [74]. The role of TIMP‐4 in tumour growth and metastasis formation in human melanoma has not yet been studied. Overall, these findings underscore the crucial role of the balance between activated MMP levels and TIMP expression in melanoma cell invasion, tumour growth and metastasis formation [71].

### 2.3 Natural Inhibitors

Apart from the synthesised and endogenous MMPIs, researchers have investigated a diverse range of natural compounds, derived from plants, microbes and marine organisms. These compounds typically exhibit natural antioxidant and anti‐inflammation properties. Scientists have explored their potential to selectively inhibit MMPs and their potential anti‐cancer effects. In comparison to synthetic counterparts, natural MMPIs tend to be more biocompatible and less toxic. The limitations associated with using synthetic MMPIs can be overcome by utilising natural substitutes to them.

## 3 MMP Inhibitors in Melanoma

Numerous studies have been conducted to assess the effectiveness of various MMPIs on melanoma. Nevertheless, to date, research efforts have predominantly concentrated on murine models with melanoma cells and/or on human melanoma cell lines. Their primary focus has been on three enzymes of the MMP family: MMP‐2, MMP‐9 and MMP‐14. To our knowledge, there have not been any clinical trials conducted on MMPIs for melanoma treatment.

MMP‐2 and MMP‐9 belong to the gelatinase class, and both are involved in the degradation of type IV and type V collagens. MMP‐2 plays a critical role in tumour invasion and metastasis.

MMP‐9 is associated with angiogenesis, inflammation and tissue remodelling. Elevated levels of MMP‐2 and MMP‐9 are often observed in various cancers [75, 76]. Unlike secreted MMPs, MMP‐14, also known as MT1‐MMP, is anchored to the cell membrane. MMP‐14 is particularly relevant in melanoma progression as it activates other MMPs, such as MMP‐2 and promotes tumour cell migration by cleaving ECM components.

Early MMPIs like Batimastat, the first generation hydroxamate‐based MMPI, were nonspecific, inhibiting virtually all MMPs. In a murine model with B16‐BL6 melanoma cells, Batimastat's efficacy was assessed, showing reduced lung metastasis and tumour size post‐cell inoculation [77]. Additionally, combining Batimastat with IL‐12 in another animal model demonstrated potent antitumoral and antiangiogenic effects [78]. In a separate study, Batimastat induced anti‐angiogenic effects in mice inoculated with B16F1 melanoma cells, significantly reducing liver metastasis size without impeding melanoma cell circulation [79]. MMI270, another hydroxamate‐based MMPI, was investigated for its impact on melanoma cell extravasation in a murine model. They suggested administering MMI270 post‐lymphadenectomy to reduce metastatic niches, hypothesising the lymphatic system as the main route of metastasis [80]. MMI‐166, a third‐generation inhibitor targeting MMP‐2 and MMP‐9, demonstrated reduced tumour proliferation in mice injected with B16‐BL6 melanoma cells. Additionally, combining MMI‐116 with paclitaxel or carboplatin enhanced its anti‐tumour efficacy [81].

Several publications have already extensively reviewed MMPIs in cancer therapy prior to 2020 [17, 18, 19, 22, 82, 83]. Here, we will focus on exploring studies conducted over the past decade that investigate synthetic and natural MMPIs in the context of melanoma treatment.

### 3.1 Synthetic MMP Inhibitors in Melanoma

Table 1 provides summaries of studies on synthetic MMPIs in relation to melanoma since 2012. Over the past decade, a wide number of MMP targeting strategies for melanoma have emerged, many showcasing the capability to reduce or inhibit specific MMP activities through direct or indirect interaction with MMPs. Consequently, they hold promise as potential therapeutic agents in melanoma treatment, as detailed in the following.

**TABLE 1:** Synthetic MMP inhibitors in melanoma.

MMPI name	Functional group	Experimental model	Year	Targeted MMPs	Results	References
Direct MMP inhibitors						
Lumican	Leucine‐rich proteoglycan	Murine B16F1 melanoma cell lines	2014	MMP‐14 (M1‐MMP)	Decreased MMP‐14 activity in B16F1 melanoma cells	[84]
ND‐322	Thiirane derivative	Mice injected with WM‐266‐4 melanoma cells	2016	MMP‐2	Inhibition of MMP2 and MMP14 activities and reduction of tumour growth and metastasis	[61]
				MMP‐14		
3A2	Human Fab antibody	Mice injected with B16F10 melanoma cells	2017	MMP‐14	Alleviated melanoma metastatic burden by inhibition of MMP‐14	[85]
SB‐3CT	Thiirane derivative	Mice injected with B16F10 melanoma cells	2020	MMP‐2	Reduced tumour burden and improved survival time by regulation of PD‐L1	[86]
				MMP‐9		
Cyclic peptide	Cyclic peptide	Human melanoma cell lines – WM115	2020	MMP‐2	Modulation of MMP‐2 activities and inhibition of cell migration by perturbing protein‐protein interactions	[87]
JR‐AB2‐011	mTORC2‐inhibitor	Human melanoma cell lines‐MelJu, MelJuSo, and MelIm, and murine B16F10 model	2020	MMP‐2	Reduced migration and invasion in human melanoma cells and reduction of tumour metastasis in murine model	[88]
JQ1	BRD4 inhibitor	Human melanoma cell lines – A375 and A375mA2	2021	MMP‐2	Prevention of melanoma cell invasion and metastasis by reducing MMP‐2 expression through inhibition of BRD4	[89]
N‐Hydroxybutanamide derivatives	N‐Hydroxy‐butanamide	Mice injected with B16 melanoma cells	2023	MMP‐2	Demonstrated antitumor and antimetastatic effects	[90]
				MMP‐9		
				MMP‐14		
Indirect MMP inhibitors						
JaZ‐30	Riazolylmethyl aziridine	Murine B16 4A5 melanoma cell line	2014	MMP‐2	Reduced melanoma cell invasion, angiogenesis and targeting ERK1/2 phosphorylation	[91]
FG‐3019	Anti‐connective tissue growth factor antibody	Mice injected with human melanoma cell	2014	MMP‐9	Reduced MMP‐9 expression and decreased melanoma cell invasion and migration	[92]
J‐4 and Celecoxib	Protein kinase C ζ and cyclooxygenase‐2 inhibitors	Mice injected with B16F10 melanoma cells	2017	MMP‐2	Inhibition of MMP‐2 and MMP‐9 expression, and suppression of melanoma metastasis	[93]
				MMP‐9		
Onconase	Ribonuclease enzyme	Human melanoma cell lines – A375 and dabrafenib‐resistant A375DR	2019	MMP‐2	Decreased MMP2 activity, and reduced cell colony formation, migration and invasion capability	[94]
Sorafenib	Tyrosine kinase inhibitor	Mice injected with B16F10 and B16F1 melanoma cells	2021	MMP‐1	Decreased expression levels of MMPs, and reduced tumour growth and enhanced survival	[95]
				MMP‐2		
				MMP‐9		
				MMP‐14		
Thiazolyl‐pyrazoline derivatives	Thiazolyl‐pyrazoline	Human melanoma A375 cell lines	2023	MMP‐2	3 out of the 11 derivatives demonstrated anti‐metastasis and anti‐inflammatory properties	[96]
				MMP‐9		

#### 3.1.1 Direct Synthetic MMP Inhibitors in Melanoma

Lumican, a specific member of the small leucine‐rich proteoglycan family, was shown to significantly decrease MMP‐14 activity in mice B16F1 melanoma cells. Acting as a competitive inhibitor, Lumican interacts directly with MMP‐14, effectively inhibiting MMP‐14 activity through binding to its catalytic domain with moderate affinity (*K*_D_ ~ 275 nM). Additionally, Lumican may protect collagen from MMP‐14 proteolysis, influencing cell‐matrix interaction in tumour progression [84].

ND‐322, a thiirane derivative with high selectivity for MT1‐MMP and MMP2, was used at concentrations that do not affect MMP9 (2.7‐fold below its Ki value). This selective inhibition effectively reduced the growth, migration and invasion of melanoma cells in vitro.

Notably, these inhibitory effects resulted in a significant reduction in melanoma tumour growth and delayed metastasis to lungs in an orthotopic mouse melanoma activity [61]. Migration and invasion appeared to be dependent on MMP2, as inhibition of this protease by specific shRNA replicated the inhibitory effects observed with ND‐322. Additionally, the introduction of recombinant active MMP2 was sufficient to rescue migration and invasion in cells in which MT1‐MMP was knocked down. These data further confirmed our previous findings that MT1‐MMP promotes melanoma metastasis by activating MMP2, which subsequently stimulates cell migration through RAC1 activation [97]. Notably, ND‐322 was able to hamper RAC1 activity, indicating that part of its inhibitory function may involve blocking the MMP2/RAC1 axis.

Recently, we reported that shRNA‐ or ND322‐mediated inhibition of MT1‐MMP synergized with BRAF inhibitor (BRAFi) leading to resensitization of resistant melanoma cells and tumours to the inhibitor [98]. Resistant cells in MT1‐MMP uncleavable matrices were resensitized to BRAFi like with direct MT1‐MMP inhibition. This resensitization is due to the disruption of ITGB1/FAK signalling, as restoration of ITGB1 activity can maintain resistance to BRAFi in the context of MT1‐MMP inhibition. Moreover, the MT1‐MMP increase in BRAFi‐resistant cells is TGFβ‐dependent, with TGFβ receptor I/II inhibition dampening MT1‐MMP levels and restoring BRAFi sensitivity [98]. Additionally, SB‐3CT, an analogue of ND‐322, exhibited high affinity for MMP‐2 and MMP‐9. Inhibition of these MMPs by SB‐3CT significantly reduced the tumour burden and improved survival time by regulating PD‐L1 in mouse models with melanoma and lung cancer [86].

A human Fab antibody (3A2) was developed by Remacle et al. [85] to specifically inhibit MT1‐MMP activity. The 3A2 Fab antibody inactivated the functional activity of cellular MT1‐MMP by binding to the catalytic domain outside the active site cavity. It inhibited cellular MT1‐MMP‐mediated collagenolysis and inhibited MT1‐MMP‐dependent cell invasion. The 3A2 competed with TIMP‐2 but not with hydroxamate inhibitor for its binding to MT1‐MMP. In addition, the 3A2 Fab reduced both the frequency and the size of melanoma metastatic nodules in mice [85].

A cyclic peptide has been reported to possess the ability to inhibit MMP‐2 activation with a low‐nanomolar potency. This inhibitor specifically bound to the D570‐A583 epitope of proMMP2 and interfered with the protein–protein interaction (PPI) between proMMP2 and tissue inhibitor of metalloproteinases‐2 (TIMP2). As a result, it prevented the TIMP2‐assisted proMMP‐2 activation process. The efficacy of this cyclic peptide in modulating cellular MMP‐2 activities and inhibiting cell migration has been confirmed in a human melanoma cell line. These findings represent the first successful example of targeting the PPI between proMMP2 and TIMP2, thus validating the feasibility of a MMP2 inhibition strategy [87].

JR‐AB2‐011, a novel mTORC2 specific inhibitor, demonstrated significant effects in melanoma. It reduced migration and invasion capacity by impairing activation of MMP‐2 in melanoma cells. Additionally, treatment with JR‐AB2‐011 led to a significant reduction in liver metastasis in a syngeneic murine metastasis model, as confirmed by in vivo imaging and necropsy. These findings highlight the potential pharmacological blockade of mTORC2 as a potent anti‐cancer strategy for liver metastasis originating from melanoma [88].

BRD4 functions as an epigenetic reader crucial for gene transcription regulation and represents a valid therapeutic target in cancer. A study by Le et al. [99] revealed that inhibiting BRD4 led to a decrease in the invasive behaviour of melanoma cells by downregulating MMP‐2 and enhances phagocytosis by myeloid cells via SIRPα downregulation [99]. Inhibiting BRD4, either through the BET pharmacological inhibitor JQ1 or BRD4‐specific siRNA, may prevent melanoma cell metastasis by curtailing cell invasion and migration while facilitating the phagocytosis‐mediated elimination of melanoma cells. The inhibition of the MMP‐2 level by siRNA resulted in a significant reduction in A375 and A375mA2 cell migration and invasion, indicating the direct involvement of MMP‐2 in melanoma invasion and/or migration. These findings hold translational significance and offer promise for melanoma patients resistant to conventional targeted therapies or immunotherapy [89].

Recently, new N‐hydroxybutanamide derivatives were synthesised using a novel method of N‐substituted succinimide ring opening. Their ability to inhibit MMPs was evaluated. The iodoaniline derivative of *N* ^1^‐hydroxy‐*N* ^4^‐phenylbutanediamide showed the inhibition of MMP‐2, MMP‐9 and MMP‐14 with an IC_50_ of 1–1.5 μM. In a murine model of B16 melanoma, this compound exhibited dual effects by significantly inhibiting tumour growth (61.5% inhibition) and metastasis (88.6% inhibition). These findings suggest that the iodoaniline derivative of N1‐hydroxy‐N4‐phenylbutanediamide holds promise as a lead structure for the development of new MMP inhibitors [90].

#### 3.1.2 Indirect Synthetic MMP Inhibitors in Melanoma

JaZ‐30, a C(2)‐monosubstituted aziridine‐aryl‐1,2,3‐triazole conjugate, was identified as a selective inhibitor of MMP‐2. JaZ‐30‐mediated decrease of vascular endothelial growth factor (VEGF) secretion resulted in inhibition of invasion and angiogenesis. Additionally, JaZ‐30 downregulated phosphorylation of the extracellular signal‐regulated kinases 1 and 2 (ERK1/2) in mitogen‐stimulated murine melanoma cells, positioning it as a potentially attractive agent for melanoma treatment [91].

Connective tissue growth factor (CTGF) expression was found to correlate with tumour progression and was strongly induced by hypoxia through HIF‐1 and HIF‐2‐dependent mechanisms in human melanoma cell lines. Inhibition of CTGF with a specific anti‐CTGF monoclonal antibody (FG‐3019) reduced MMP‐9 expression and significantly impeded the ability of human melanoma cells to grow in the skin. Furthermore, it prevented the establishment of distant metastases in the lungs of SCID mice. FG‐3019 has undergone preclinical validation as a promising anti‐CTGF therapy for advanced melanoma [92].

Melanoma metastasis correlates with the heightened activation of protein kinase C ζ (PKCζ) and cyclooxygenase‐2 (COX‐2) signalling pathways. Inhibition of both PKCζ and COX‐2 shows promise for suppressing melanoma metastasis. Zhou et al. [93] combined the PKCζ inhibitor J‐4 with the COX‐2 inhibitor Celecoxib to treat melanoma metastasis. They found that this combination synergistically inhibited migration, invasion and adhesion of melanoma cells (B16F10 and A375) in vitro. Moreover, secretion of MMP‐2 and MMP‐9 was significantly reduced by the combined treatment. In C57BL/6 mice intravenously injected with B16F10 cells, the co‐treatment of J‐4 and Celecoxib also significantly suppressed melanoma lung metastasis [93].

Onconase, a cytotoxic RNase variant, was studied for its efficacy against BRAFi‐resistant cells, aiming to restore the efficacy of the chemotherapy. Onconase affected the total viability and the proliferation rate in both parental (A375) and the dabrafenib‐resistant (A375DR) human melanoma cells in a dose‐dependent manner. Additionally, Onconase reduced the levels of nuclear p65 NF‐κB and IKK phosphorylation and decreased MMP‐2 activity in both cell types. It also inhibited cell colony formation, migration and invasion, suggesting potential for combating melanoma recurrence, particularly in BRAFi‐resistant cells [94].

Sorafenib is a tyrosine kinase inhibitor that targets key pathways in cancer progression. It has been reported to inhibit B‐RAF, c‐RAF, platelet derived growth factor receptor (PDGFR), VEGFR and c‐Kit [100]. In a study conducted by Takeda et al. [95], the effect of sorafenib on metastatic melanoma with c‐Kit aberration was examined in a murine model. Sorafenib was found to reduce mRNA expression and enzymatic activities of MMP‐1, MMP‐2, MMP‐9 and MMP‐14 in a concentration‐dependent manner. Additionally, it decreased cell viability, migration and invasion in vitro, and inhibited metastasis and tumour growth in vivo. Sorafenib treatment notably enhanced overall survival in mice, indicating its potential as a therapeutic option for melanoma with c‐Kit dysregulation [95].

Hosseini et al. [96] synthesised 11 thiazolyl‐pyrazoline derivatives, known for their diverse activities, as potential anticancer compounds. These derivatives were evaluated for their anti‐proliferative activities against human lung carcinoma and human melanoma (A375) cell lines in vitro. Promising compounds were further investigated for their anti‐metastasis and anti‐inflammatory properties through inhibition of the expression of MMP‐2, MMP‐9 and cyclooxygenase 2. Three derivatives demonstrated significant potential for future research in developing novel anticancer agents [96].

### 3.2 Direct Natural MMP Inhibitors in Melanoma

Table 2 outlines research conducted on natural MMPIs in relation to melanoma since 2012. There has been a notable increase in studies exploring the effects of natural MMPIs on melanoma in recent years. Many natural compounds demonstrate the ability to inhibit cell proliferation, growth, invasion and migration while exhibiting anti‐angiogenic, anti‐inflammatory and anticancer properties. These attributes strongly indicate the potential of natural MMPIs in melanoma treatment, as elaborated upon below.

**TABLE 2:** Natural MMP inhibitors in melanoma.

MMPI name	Product source	Experimental model	Year	Targeted MMPs	Results	References
Direct MMP inhibitors						
SIP‐SII	Sulfated Sepiella maindroni ink polysaccharide	Mice injected with B16F10 melanoma cells	2013	MMP‐2	Antimetastatic activity against B16F10 melanoma	[101, 102]
Eupatorium fortunei	Herbal medicine	Mice injected with B16F10 melanoma cells	2014	MMP‐9	Suppressed metastatic properties by downregulating proteolytic activity of MMP9	[103]
Nano‐encapsulated Curcumin and Chrysin	Curcumin and Chrysin	Mice bearing B16F10 melanoma tumour	2018	MMP‐2	Decreased MMP‐2 and MMP‐9 expression, and inhibition of tumour Growth	[104]
				MMP‐9		
Laminaran and its sulfated derivative	Water‐soluble *β‐D‐glucans of brown algae*	Human melanoma SK‐MEL‐28 cells	2019	MMP‐2	Inhibition of MMP‐2 and MMP‐9 proteinases activity and down‐regulation of kinases’ phosphorylation of ERK1/2 signalling cascade	[105]
				MMP‐9		
Ethanol extract of Angelica dahurica Radix	*Angelica dahurica Radix*	Mice injected with B16F10 melanoma cells	2020	MMP‐2	Suppression of tumour growth and metastasis through inhibiting of MMP‐2 and ‐9 expression	[106]
				MMP‐9		
22*β*‐hydroxytingenone	*Salacia impressifolia A.C. Smith*	Human melanoma cell line‐SK‐MEL‐28	2021	MMP‐9	Suppressed invasiveness of melanoma cells by inhibiting MMP‐9 activity and MAPK signalling	[107]
Indirect MMP inhibitors						
Oroxylin A	*Scutellaria radix*	Mice injected with B16F10 melanoma cells	2012	MMP‐2	Reduced MMP‐2 and MMP‐9 activity and inhibited lung metastasis of melanoma cell	[108]
				MMP‐9		
Bornyl *cis*‐4‐hydroxycinnamate	*Piper betle* stems	Human melanoma cells – A375	2018	MMP‐2	Reduced MMP‐2 and MMP‐9 expression through inhibition of FAK/PI3K/Akt/mTOR, MAPK, and GRB2 signalling pathways	[109]
				MMP‐9		
Oxyfadichalcone C	*Oxytropis falcate*	Human melanoma cell lines – A375	2018	MMP‐2	Inhibition of cell proliferation and metastasis via suppression PI3K/Akt and MAPK/ERK Pathways	[110]
				MMP‐9		
Herbacetin	Flaxseed, *Sinocrassula indica*, and *Rhodiola crenulata*	Human melanoma cell lines ‐ A375 and Hs294T, and Mice injected with A375	2019	MMP‐9	Suppressed MMP9 mediated angiogenesis of melanoma through blocking EGFR‐ERK/AKT signalling pathway	[111]
Emodin	*Rhubarb roots* and *rhizomes*	Human melanoma (A375) and mouse B16F10 cell lines	2021	MMP‐2	Decreased MMP‐2 and MMP‐9 protein expression, and inhibition of cell proliferation	[112]
				MMP‐9		
Asiatic acid + Naringenin	*Centella asiatica* Citrus fruits	Mice inoculated with B16F10 melanoma cells	2021	MMP‐2	Suppression of tumour invasion and metastasis by targeting TGF‐*β*‐MMP2 pathway	[113]
Dioscin	*Dioscorea opposita* Thunb	B16F10 melanoma allograft mice	2022	MMP‐2	Restrained melanoma growth by inhibiting Src/STAT3 signalling‐mediated angiogenesis	[114]
				MMP‐9		

#### 3.2.1 Direct Natural MMP Inhibitors in Melanoma

SIP‐SII, a sulfated *Sepiella maindroni* ink polysaccharide, inhibited cancer cell invasion and migration by blocking MMP‐2 proteolytic activity [101]. Zong et al. [102] investigated its anti‐metastatic effect of SIP‐SII in a melanoma mouse model, administering doses of 15 and 30 mg/kg/day. Remarkably, SIP‐SII significantly reduced B16‐F10 pulmonary metastasis by 85.9% and 88.0%, respectively. Immunohistochemistry analysis showed SIP‐SII downregulated the expression of intercellular adhesion molecule 1 (ICAM‐1) and basic fibroblast growth factor (bFGF) in lung metastasis nodules, suggesting it inhibits tumour adhesion and angiogenesis, as well as cancer cell invasion and migration [102].

Eupatorium fortunei is traditionally used to alleviate nausea and enhance appetite, and is prescribed in Chinese medicine as a diuretic and detoxifier. Kim et al. [103] examined an aqueous extract of *E. fortunei* (WEF), finding it inhibited metastasis and angiogenesis of malignant tumour cells both in vitro and in vivo. WEF inhibited colony formation, migration and invasion of tumour cells through the downregulation of MMP‐9 proteolytic activity. It also markedly reduced NF‐kB activation, p38 and JNK phosphorylation. In a murine pulmonary metastasis model with B16F10 melanoma cells, daily WEF administration decreased metastatic colonies, suggesting its potential as a therapeutic herb for metastatic cancer [103].

Two natural anti‐cancer compounds, Curcumin and Chrysin, co‐encapsulated in PLGA‐PEG nanoparticles (NPs), were studied in a murine model bearing B16‐F10 melanoma tumours. NPs led to a significant decrease in MMP‐2, MMP‐9 and TERT gene expression compared to the control. Conversely, TIMP‐1 and TIMP‐2 gene expression was notably increased in the NPs‐treated group compared to the control. Furthermore, NPs treatment significantly inhibited melanoma tumour growth. These results suggest that the nano‐combination of Curcumin and Chrysin into polymeric NPs, utilising a one‐step fabricated co‐delivery system, holds promise as a convenient and effective strategy to enhance their efficacy in melanoma therapy [104].

Laminarans, β‐d‐glucans of brown algae, possess potent immunomodulating, radioprotective and anticancer activities. Both native and sulfated laminarans showed significant anticancer activity against melanoma cells at non‐toxic doses. While both polysaccharides protected normal epidermal cells, only sulfated laminaran sensitised melanoma cells to x‐ray irradiation, resulting in a notable inhibition of cell proliferation, colony formation and cancer cell migration. This occurred via MMP‐2 and MMP‐9 inhibition and ERK1/2 signalling downregulation. Combining the sulfated laminaran derivative with X‐ray radiation offers a probable melanoma treatment strategy [105].

Ethanol extracted from the root of *Angelicae dahuricae Radix* (EEAD) effectively inhibited cell growth and induced apoptosis in B16‐F10 cells. It also reduced cell migration, invasion and colony formation by targeting MMP‐2 and MMP‐9. In vivo studies demonstrated EEAD's ability to mitigate lactate dehydrogenase activity, hepatotoxicity and nephrotoxicity in B16‐F10 cell‐inoculated mice. Additionally, EEAD significantly suppressed lung hypertrophy, B16‐F10 cell lung metastasis and tumour necrosis factor‐alpha expression in lung tissue. These discoveries imply EEAD's capability for managing metastasis and growth in malignant melanoma [106].

22β‐hydroxytingenone (22‐HTG), a quinonemethide triterpene derived from *Salacia impressifolia* (Miers) A. C. Smith, was investigated for its ability to induce apoptosis and inhibit metastasis in SK‐MEL‐28 human melanoma cells. Results showed that 22‐HTG possessed anti‐tumorigenic properties against melanoma cells by inducing cell cycle arrest and apoptosis while inhibiting invasiveness potential, as demonstrated in a 3D model of reconstructed human skin. Moreover, 22‐HTG induced apoptosis and suppressed melanoma cell invasiveness by inhibiting MMP‐9 activity and MAPK signalling pathways [107].

#### 3.2.2 Indirect Natural MMP Inhibitors in Melanoma

Flavonoids, known for their anti‐inflammatory and anticancer properties, exhibit promising therapeutic potential. Oroxylin A, from *Scutellaria* roots, was found to suppress cell adhesion, invasion and migration in vitro. It modulated proteolytic activity, downregulated MMP‐2 and MMP‐9 and upregulated TIMP‐2 gene expression. Oroxylin A inhibited PMA‐induced protein kinase C translocation and extracellular signal‐regulated kinase phosphorylation and attenuated activator protein‐1 binding activity. These actions targeted the upstream signalling molecules involved in MMP‐9 expression. In vivo, Oroxylin A inhibited lung metastasis of murine melanoma cell B16F10 [108]. Another flavonoid compound, Herbacetin, from flaxseed and other sources exhibited promising anti‐angiogenic effects in human malignant melanoma by blocking EGFR‐ERK/AKT‐MMP9 signalling pathway. Both compounds show promise for melanoma treatment [111].

Bornyl cis‐4‐hydroxycinnamate, from *Piper betle* stems, has emerged as an encouraging agent for inhibiting melanoma cell migration and invasion. It modulated melanoma cell metastasis by reducing MMP‐2 and MMP‐9 expression through the inhibition of the FAK/PI3K/Akt/mTOR, MAPK and GRB2 signalling pathways. Additionally, bornyl cis‐4‐hydroxycinnamate impeded the epithelial‐to‐mesenchymal transition process in human A375 melanoma cells [109].

Oxyfadichalcone C, a chalcone dimer derived from Oxytropis falcata, was studied for its in vitro effects on human A375 cells. It showed significant anti‐proliferative properties by inducing G1 phase arrest and mild apoptosis. Additionally, it exhibited notable anti‐migration and anti‐invasion capabilities. These effects correlated with upregulated levels of p27 and decreased expression of cyclin D1, p‐pRb, p‐Integrin β1, as well as the activity of MMP‐2 and MMP‐9. Furthermore, downregulation of key molecules in the PI3K/Akt and MAPK/ERK pathways suggested their involvement in Oxyfadichalcone C's inhibition of proliferation and metastasis in A375 cells. Combined with Vemurafenib at a 1:5 ratio, Oxyfadichalcone C demonstrated synergistic anti‐proliferative effects, highlighting its potential in melanoma treatment [110].

Emodin, found in rhubarb roots and rhizomes, demonstrates anti‐tumour effects. Studies on mice B16‐F10 and human A375 melanoma cells have shown that it inhibited cell proliferation and induced apoptosis. Immunofluorescence and western blot analysis confirmed reduced MMP‐2 and MMP‐9 protein expression and an increased Bax/Bcl‐2 ratio following emodin treatment, which indicates emodin's potential as a therapeutic option for highly metastatic melanoma [112].

Lian et al. [113] discovered that combining Asiatic acid (AA), a Smad7 agonist from *Centella asiatica* , with Naringenin (NG), a Smad3 inhibitor mainly from citrus fruits successfully rebalanced Smad3 and Smad7 signalling in TGF‐β‐rich tumour microenvironment. This strategy notably curbed tumour invasion and metastasis in mouse models of melanoma and lung carcinoma. Treatment with NG reduced Smad3‐mediated MMP‐2 transcription and increased TIMP2 expression, while AA enhanced Smad7 to suppress TGF‐β/Smad3 signalling and MMP‐2 activation by targeting the NF‐κB‐MT1‐MMP axis. Together, AA and NG effectively suppressed invasion and metastasis of melanoma and lung carcinoma by modulating TGF‐β/Smad‐dependent MMP2 transcription, post‐translational activation and function [113].

Dioscin, a steroid saponin found in diverse plants like *Dioscorea opposita Thunb*, has demonstrated the capability to impede melanoma growth and angiogenesis in B16‐F10 allograft mouse models [115]. It operated by suppressing Src and STAT3 activation, reducing mRNA and protein levels of STAT3‐regulated genes. Furthermore, dioscin decreased the secretion of MMP‐2, MMP‐9 and VEGF from melanoma cells treated with dioscin. The inhibition of Src/STAT3 signalling‐mediated angiogenesis contributed significantly to the anti‐melanoma effects of dioscin [114].

## 4 Conclusion and Prospect

Numerous preclinical and clinical studies highlight the significant inhibitory potential of MMPIs in reducing melanoma tumour growth and metastasis. It was hypothesised that MMP inhibition could effectively curtail tumour progression during its early stages [18]. Although the efficacy of MMPI therapy may vary as the disease advances, ongoing research is continuously revealing new insights and strategies to optimise their therapeutic application. While early clinical trials with broad‐spectrum MMP inhibitors demonstrated limited success and notable side effects, selective MMP inhibitors provide a more targeted approach, potentially overcoming these issues. By targeting specific MMPs that are essential for melanoma invasion, metastasis and angiogenesis while avoiding those that may have beneficial roles in normal physiology, selective inhibitors could enhance treatment efficacy and minimise off‐target effects observed with earlier broad‐spectrum therapies. In addition, combining MMP inhibitors with other targeted therapies, such as BRAF inhibitors, or immunotherapies, such as checkpoint inhibitors, shows considerable promise. This combination approach has the potential to synergistically enhance treatment efficacy by disrupting ECM remodelling, overcoming resistance mechanisms and improving immune responses against melanoma cells.

Natural MMPIs stand out as a promising source of anti‐melanoma compounds due to their biocompatibility and fewer side effects. Many natural MMP inhibitors exhibit multifaceted properties. They not only inhibit MMPs but also demonstrate antioxidant, anti‐inflammatory and immune‐modulatory effects. This dual action can enhance their therapeutic potential and improve overall cancer treatment. The development of bioactive natural compounds into drugs is indeed a challenging journey, involving large‐scale isolation, mechanistic understanding and pharmaceutical development. However, recent technological advancements and a deeper understanding of cancer have significantly boosted interest in natural products as potential therapeutic agents. Natural MMPIs hold the potential to complement existing therapies, enhancing pharmacological efficacy and reducing toxicity. Researchers continue to explore these compounds, aiming to rejuvenate anticancer drug discovery and augment treatment modalities for melanoma and other malignancies.

## Author Contributions

E.Z. conceived the manuscript structure, conducted the literature review and wrote the manuscript; V.T. and B.B. supervised, reviewed and edited the manuscript.

## Funding

The authors have nothing to report.

## Conflicts of Interest

The authors declare no conflicts of interest.
